# Association between chronic periodontitis and the risk of Alzheimer’s disease: combination of text mining and GEO dataset

**DOI:** 10.1186/s12903-021-01827-2

**Published:** 2021-09-23

**Authors:** Zhengye Jiang, Yanxi Shi, Wenpeng Zhao, Liwei Zhou, Bingchang Zhang, Yuanyuan Xie, Yaya Zhang, Guowei Tan, Zhanxiang Wang

**Affiliations:** 1 Department of Neurosurgery, Xiamen Key Laboratory of Brain Center, The First Affiliated Hospital of Xiamen University, Xiamen, China; 2 The Department of Neuroscience, Institute of Neurosurgery, School of Medicine, Xiamen University, Xiamen, China; 3 Department of Cardiology, Jiaxing Second Hospital, Jiaxing, China

**Keywords:** Differentially expressed genes, Chronic periodontitis, Alzheimer disease, Signaling pathway

## Abstract

**Background:**

Although chronic periodontitis has previously been reported to be linked with Alzheimer's disease (AD), the pathogenesis between the two is unclear. The purpose of this study is to analyze and screen the relevant and promising molecular markers between chronic periodontitis and Alzheimer's disease (AD).

**Methods:**

In this paper, we analyzed three AD expression datasets and extracted differentially expressed genes (DEGs), then intersected them with chronic periodontitis genes obtained from text mining, and finally obtained integrated DEGs. We followed that by enriching the matching the matching cell signal cascade through DAVID analysis. Moreover, the MCODE of Cytoscape software was employed to uncover the protein–protein interaction (PPI) network and the matching hub gene. Finally, we verified our data using a different independent AD cohort.

**Results:**

The chronic periodontitis gene set acquired from text abstracting was intersected with the previously obtained three AD groups, and 12 common genes were obtained. Functional enrichment assessment uncovered 12 cross-genes, which were mainly linked to cell morphogenesis involved in neuron differentiation, leading edge membrane, and receptor ligand activity. After PPI network creation, the ten hub genes linked to AD were retrieved, consisting of SPP1, THY1, CD44, ITGB1, HSPB3, CREB1, SST, UCHL1, CCL5 and BMP7. Finally, the function terms in the new independent dataset were used to verify the previous dataset, and we found 22 GO terms and one pathway, "ECM-receptor interaction pathways", in the overlapping functional terms.

**Conclusions:**

The establishment of the above-mentioned candidate key genes, as well as the enriched signaling cascades, provides promising molecular markers for chronic periodontitis-related AD, which may help the diagnosis and treatment of AD patients in the future.

## Background

Periodontitis constitutes a chronic inflammatory disease. During the development of periodontitis, associated complications such as alveolar bone destruction, as well as the loss of attachment of collagen fibers to periodontal ligament, will occur, eventually leading to tooth loss [1]. There are reports that the occurrence of chronic periodontitis may be related to the increase of IL-6 [2]. At the same time, interdisciplinary disease studies have shown that the serum and saliva levels of Galectin-3 in patients with chronic periodontitis + coronary heart disease (CHD) are significantly higher than those in patients with just CHD [3]. The concentration the concentration of serum and saliva NLRP3 in patients with chronic periodontitis + type-II diabetes mellitus (DM) is also significantly higher than that of patients with simple type-II DM [4]; results indicated that periodontitis was significantly correlated with the above biomarkers. However, in the studies on chronic periodontitis and neurodegenerative diseases such as cognitive decline, although there have been relevant reports, such as Cestari et al. 's results showing that the level of inflammatory cytokines in individuals with Alzheimer's disease (AD) is correlated with periodontitis, it is still unclear which specific gene targets are involved [5].

AD constitutes a progressive neurodegenerative disease. Its clinical indications primarily include cognitive decline, which eventually develops into AD. It has a place in diseases that threaten the lifespan of the elderly. A large number of previous studies have confirmed that immune factors, depression, genetic factors, etc. could be positively correlated with the incidence and development of AD [6–11]. Despite the huge advances in AD research, the current AD treatments can only improve and relieve patient conditions to some level [12]. As the threat of AD to the elderly becomes greater and greater, it is imperative for us to establish the etiology, as well as the molecular features of AD disease.

At present, high-throughput sequencing techniques, such as molecular diagnosis, prognosis estimation, as well as drug target discovery, which can be employed to assess the gene expression differences, as well as the variable splicing variation, are gradually considered to have important clinical significance in disease research. The Integrated Gene Expression Database (GEO), a publicly available website supported by the National Center for Biotechnology Information (NCBI), harbors dozens of basic experimental disease gene expression patterns and is extensively employed to explore key genes and prospective mechanisms of disease onset and development [13]. Though the pathogenesis of chronic periodontitis has been recently found to be related to AD, its pathogenesis, as well as the molecular mechanism, remains unknown. Hence, we need to utilize the gene expression chip in the bulletin database and explore its data via modern software to find novel diagnostic biomarkers and treatment targets [14].

Herein, we retrieved GSE5281, GSE15222 and GSE132903, the human AD gene expression patterns, respectively, from the GEO website. After that, R software (V. 3.6.3) installed Limma package was utilized to screen the differentially expressed genes (DEGs) [15, 16]. Text mining about chronic periodontitis was then carried out by the pubmed2ensembl online tool [17]. After the data obtained from microarray, and the text mining, were intersected to obtain the common gene, GO enrichment and KEGG pathway assessment were performed on the obtained DEGs [18]. Then, the PPI (protein–protein interaction) network was developed using the Search Tool for the Retrieval of Interacting Genes (STRING), along with Cytoscape software, to screen candidate hub genes, as well as the highly relevant functional modules. Finally, we verified our results using a different independent GSE28146 cohort. From these findings, we could find the gene biomarkers and linked cascades that might be linked to AD, providing novel insights into the molecular mechanism underlying hidden AD. In short, we explore the molecular biomarkers by studying the correlation between chronic periodontitis and AD disease to provide evidence for early diagnosis, prevention, and treatment of this disease.

## Methods

### Data abstraction

We retrieved the gene expression chip data GSE5281, GSE15222, GSE132903 and GSE28146 from the NCBI GEO data repository (https://www.ncbi.nlm.nih.gov/geo/) [13, 19]. These four cohorts all contained ten control samples and ten AD samples.

### Identification of DEGs

The core R package was employed to process the abstracted matrix files. Following the normalization, we determined the differences between AD and the control group via truncation criteria (|log fold change (FC)|≥ 1, adjusted *P* < 0.05), and determined the significant DEGs for subsequent analyses [20].

### Text mining

We carried out the text mining based on the pubmed2ensembl public tool (http://pubmed2ensembl.ls.manchester.ac.uk/). When manipulated, pubmed2ensembl retrieves all the gene names from the existing literature relevant to the research topic. We screened for chronic periodontitis. We then uncovered all the genes linked to the topic from the data. Finally, we used the gene set acquired by text mining and the previously abstracted differential gene set for the subsequent step of analysis after the intersection.

### Gene ontology analysis of DEGs, along with KEGG pathway analysis

The obtained DEGs were imported to David V. 6.8 (https://david.ncifcrf.gov/). The GO annotation, along with KEGG cascade enrichment, were carried out in the web resource, which provided a sequence of functional annotation tools for systematic analysis of biological significance of gene lists. The above gene tables were analyzed with adjusted *P* < 0.05 as the significant threshold.

### Assessment of the PPI network of the DEGs

We used the STRING online search tool to analyze the PPI data encoded by DEGs [21], and only the combination score > 0.6 was considered significant. Then, the PPI network was analyzed and visualized using Cytoscape, and the first five hub genes were determined as per the connectivity between DEGs. The standard default setting of the mcode parameter. The function enrichment of DEGs of each module was analyzed by adjusted *P* < 0.05 as the cutoff standard.

### Drug-gene usually: crosstalk and functional analysis of potential genes

The drug gene interaction database (DGIDB) was used to screen potential drug delivery targets for mutated and altered genes [22].

### Statistical analysis

Statistical analysis was performed using R/BioConductor (R Foundation for Statistical Computing, version 3.6.3). All indicated *p *values are two*-*tailed values*. p* < 0.05 was considered significant.

## Results

### DEGs identification

**Fig. 1:**
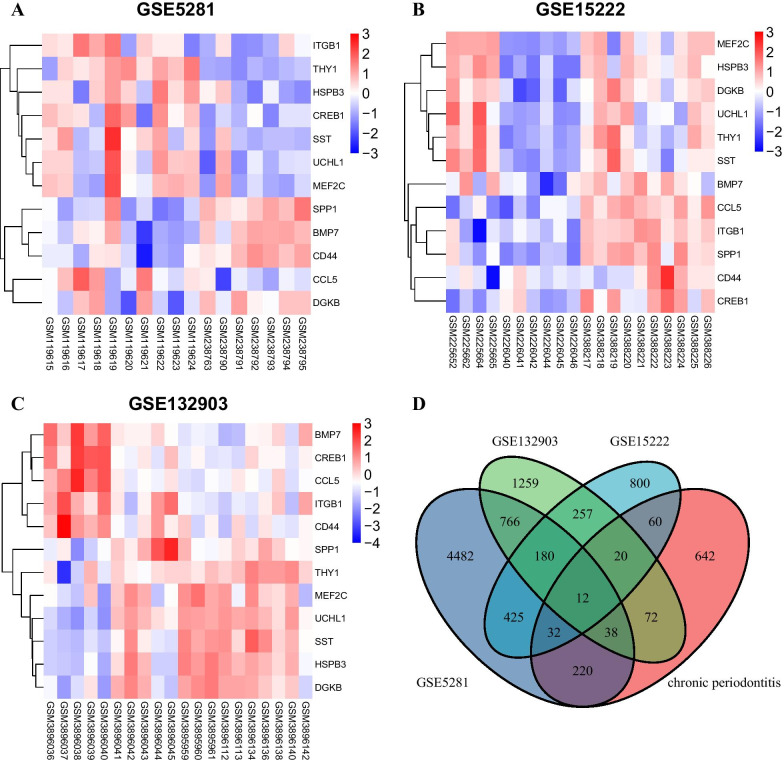
Twelve DEGs were identified by integrated analysis of AD gene expression datasets. **a**–**c** Clustering of the 12 DEGs in AD versus control across each independent dataset. Each column represents a sample and each row represents the expression level of a given gene. The color scale represents the raw Z score ranging from blue (low expression) to red (high expression). Dendrograms by each heatmap correspond to the hierarchical clustering by expression of the 12 genes. **d** Venn diagram of DEGs from three microarray datasets and genes list from text mining

Firstly, we selected 6155 DEGs from AD samples and healthy controls in the GSE5281 data set via limma package screening of R software. Of these, we selected 2201 upregulated genes and 3954 downregulated genes. At the same time, 1787 DEGs consisting of 1431 upregulated genes and 355 downregulated genes, were uncovered via analysis of the AD samples in the GSE15222 data set. And from the GSE132903 dataset, we also obtained 1303 upregulated genes and 1301 downregulated genes. Then, the overall distribution of the three data sets and the first 12 DEGs were represented by volcano map, and heat map respectively (Fig. 1a–c), using |log FC|≥ 1 criteria and adjusted P < 0.05.

Through text abstraction, 1096 human genes were linked to chronic periodontitis (S. s 1). After crossing the DEGs in the microarray data, the intersection of chosen genes was determined, and 12 genes participating in AD group were obtained (Fig. 1d).

### Function along with signal cascade enrichment analysis

**Fig. 2:**
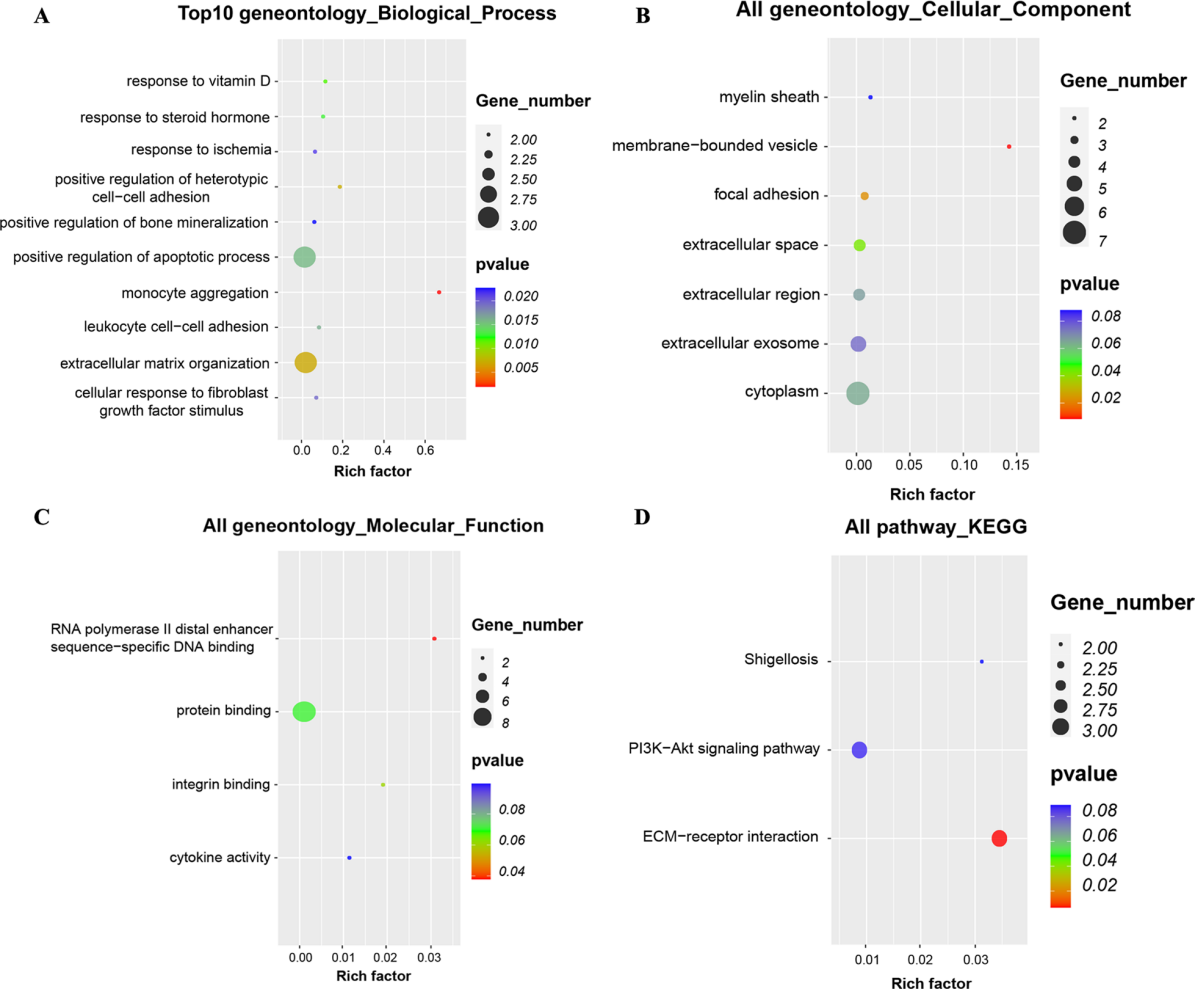
All available significant gene ontology enrichment terms and signal pathway of the common genes from three datasets and text mining. **a**–**c** A Top 10 GO terms. Number of gene of GO analysis was acquired from DAVID functional annotation tool. P < 0.05. D KEGG pathway

After introducing the DEGs obtained above into DAVID, we subjected them to GO and KEGG enrichment analysis. GO term assessment illustrated that these genes, which were abundant in cell morphogenesis, were involved in neuron differentiation (BP), leading edge membrane (CC), and receptor ligand activity (MF) (Fig. 2a–c), respectively. KEGG cascade analysis identified 3 pathways associated with the DEGs: ECM − receptor interaction, PI3K − Akt signaling cascade, and shigellosis (Fig. 2d).

### Module screening from the PPI network

**Fig. 3:**
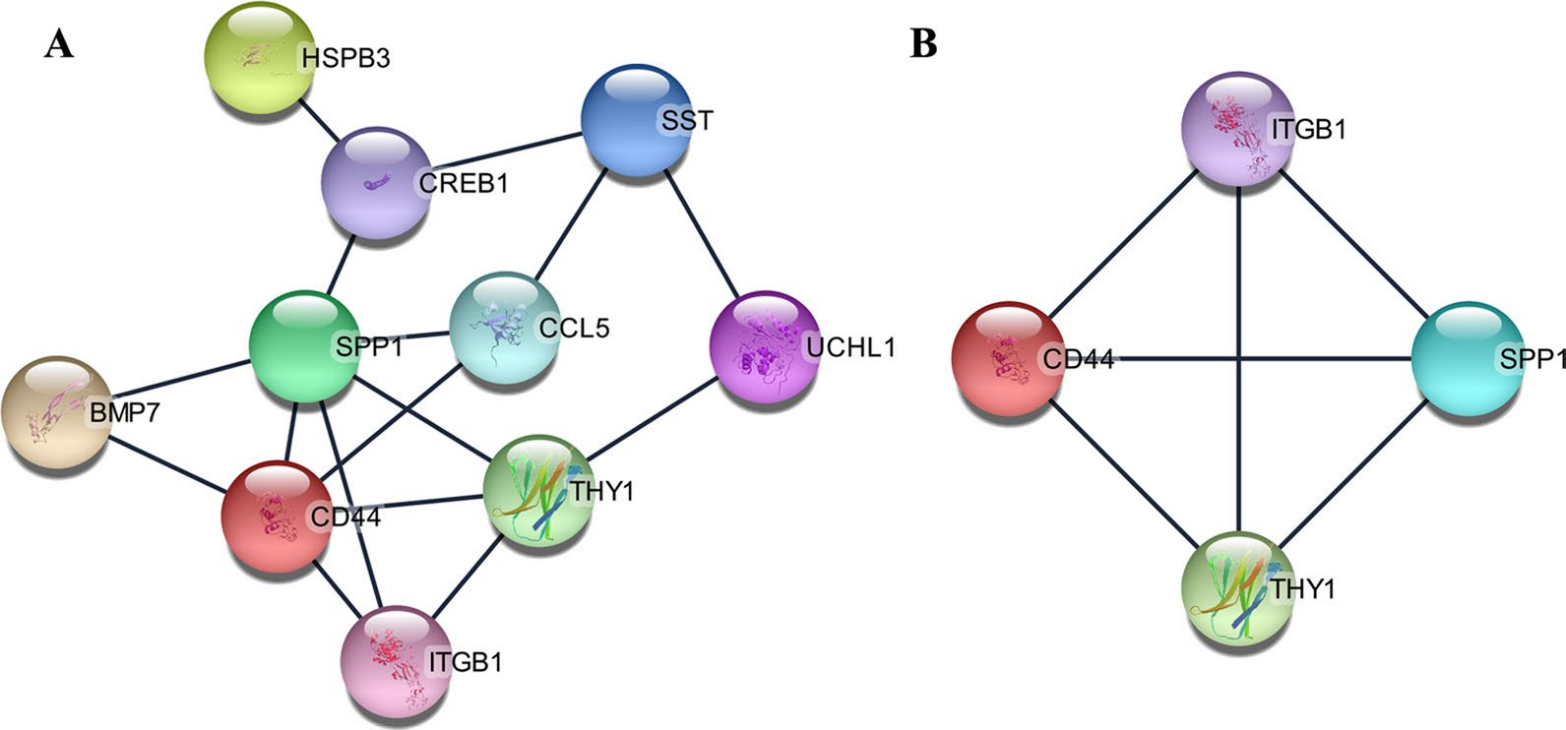
The protein–protein interaction (PPI) networks construction and significant gene modules analysis. **a** Based on the STRING online database, 12 common genes were filtered into common genes PPI network. **b** The most significant module from the PPI network

Based on the 12 co-genes, the Cytoscape publicly available platform and the STRING resource were employed to develop the PPI network, perform module analysis, as well as visualization. Consequently, we developed a PPI network bearing 16 crosstalk based on 10 integrated DEGs related to AD (Fig. 3a). We employed the MCODE algorithm to determine highly interconnected subnets, which are frequently protein complexes, as well as components of cascades as per the topological structure. We selected only one module from the entire network for further analysis (Fig. 3b). Additional functional enrichment assessment of the established modules demonstrated that genes in the module were majorly abundant in the GO, in terms of “extracellular matrix organization”, “focal adhesion”, “integrin binding”, as well as KEGG cascade of “ECM-receptor interaction” (Table 1).

**Table 1:** Functional enrichment assessment of the established modules

Term	Category	Category
GO:0030198	BP	Extracellular matrix organization
GO:0007155	BP	Cell adhesion
GO:0022617	BP	Extracellular matrix disassembly
GO:0007160	BP	Cell–matrix adhesion
GO:0016337	BP	Single organismal cell–cell adhesion
GO:0050900	BP	Leukocyte migration
GO:0043547	BP	Positive regulation of GTPase activity
GO:0005925	CC	Focal adhesion
GO:0070062	CC	Extracellular exosome
GO:0045121	CC	Membrane raft
GO:0009897	CC	External side of plasma membrane
GO:0009986	CC	Cell surface
GO:0048471	CC	Perinuclear region of cytoplasm
GO:0005178	MF	Integrin binding
hsa04512	KEGG	ECM-receptor interaction
hsa05131	KEGG	Shigellosis
hsa04670	KEGG	Leukocyte transendothelial migration
hsa05205	KEGG	Proteoglycans in cancer
hsa04510	KEGG	Focal adhesion

*GO* gene ontology, *BP* biological processes, *CC* cellular composition, *MF* molecular function, *KEGG* Kyoto Encyclopedia of Genes and Genomes

### Verification in GSE28146 cohort

**Fig. 4:**
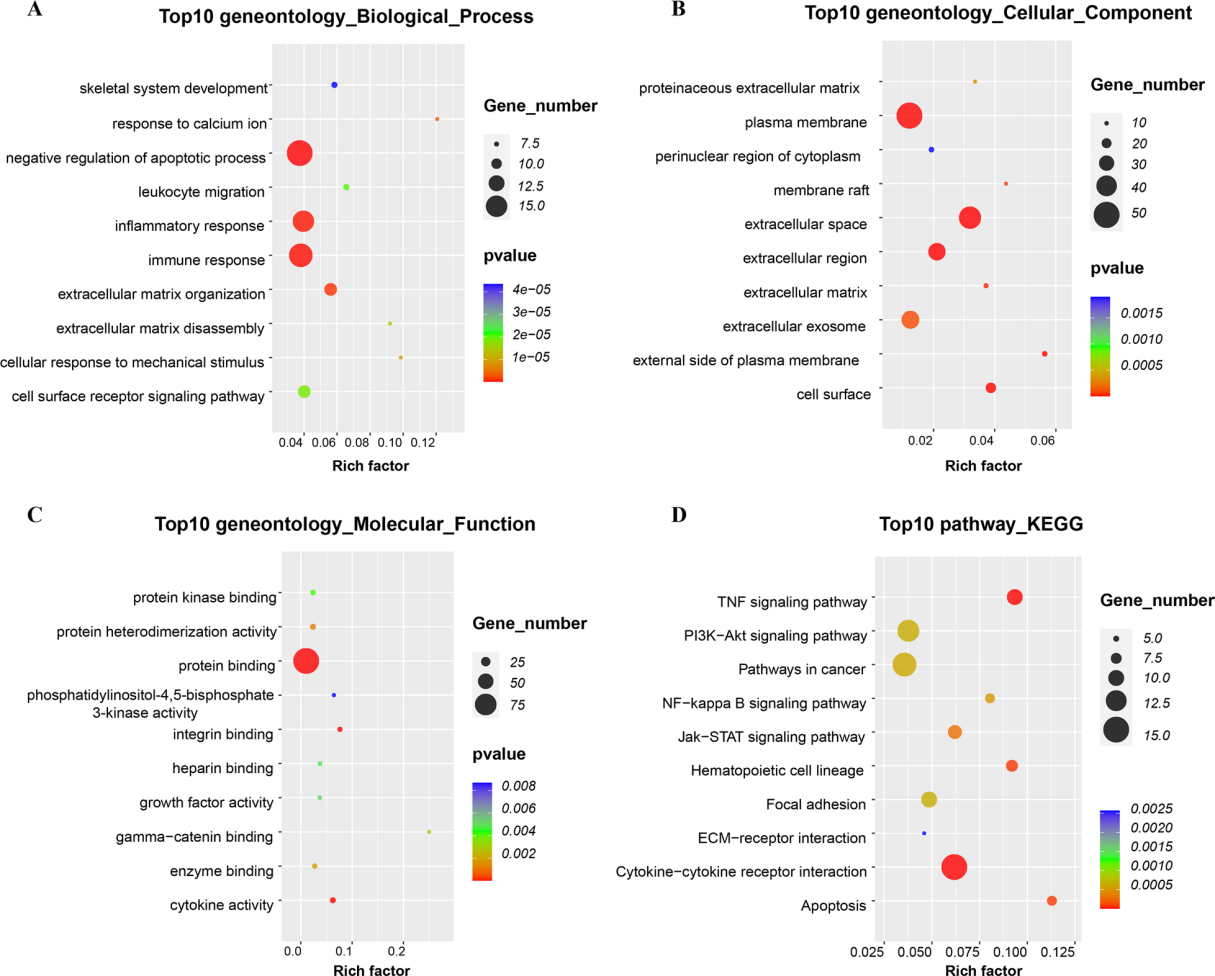
All available significant gene ontology enrichment terms and signal pathway of the common genes from GSE28146 dataset. **a–c** Top 10 GO terms. Number of gene of GO analysis was acquired from DAVID functional annotation tool. P < 0.05. D KEGG pathway

To assess the reliability of the findings derived from previous cohort, we extracted a cohort of ten AD samples and ten healthy control samples from a different independent AD dataset, GSE28146, and analyzed its gene expression data (Fig. 4). Interestingly, we found an enriched feature overlap between GSE28146 and the previous data set: there were 22 GO terms in the overlapping functional terms. And it is worth noting that when we added the gene enrichment analysis of the modules together, we found in KEGG there was only one pathway, "ECM-receptor interaction" (Table 2).

**Table 2:** Overlap of the enriched function terms between the two datasets

Term	Category	Category
GO:0070487	BP	Monocyte aggregation
GO:0030198	BP	Extracellular matrix organization
GO:0034116	BP	Positive regulation of heterotypic cell–cell adhesion
GO:0043065	BP	Positive regulation of apoptotic process
GO:0044344	BP	Cellular response to fibroblast growth factor stimulus
GO:0043407	BP	Negative regulation of MAP kinase activity
GO:0007155	BP	Cell adhesion
GO:0045669	BP	Positive regulation of osteoblast differentiation
GO:0045893	BP	Positive regulation of transcription, DNA-templated
GO:0022617	BP	Extracellular matrix disassembly
GO:0045666	BP	Positive regulation of neuron differentiation
GO:0005925	CC	Focal adhesion
GO:0005615	CC	Extracellular space
hsa04512	KEGG	ECM-receptor interaction

*GO* gene ontology, *BP* biological processes, *CC* cellular composition, *MF* molecular function, *KEGG* Kyoto Encyclopedia of Genes and Genomes

### Drug-gene crosstalk and functional analysis of potential genes

Using the DGIDB data resource, we analyzed drug-gene interactions among four potential genes aggregated in key gene modules. As a result, six drugs interacted with the gene SPP1, five also interacted with CD44, and ITGB1 was closely associated with nine different drugs. Among the 20 drugs discovered, 7 drugs (Calcitonin, Wortmannin, Gentamicin, Tacrolimus, Progesterone, Gentamicin, and Hyaluronan) have been reported to have certain experimental and clinical use for the treatment of AD. The remaining 13 drugs have not been found to be related to the treatment of AD and can be used as potential target drugs for AD (Table 3).

**Table 3:** Candidate drugs targeting genes with AD

Number	Drug	Gene
1	**ASK-8007**	SPP1
2	CALCITONIN	SPP1
3	**ALTEPLASE**	SPP1
4	WORTMANNIN	SPP1
5	GENTAMICIN	SPP1
6	TACROLIMUS	SPP1
7	PROGESTERONE	CD44
8	**BIVATUZUMAB**	CD44
9	**HYALURONATE SODIUM**	CD44
10	GENTAMICIN	CD44
11	HYALURONAN	CD44
12	**ABITUZUMAB**	ITGB1
13	**VOLOCIXIMAB**	ITGB1
14	**NATALIZUMAB**	ITGB1
15	**INTETUMUMAB**	ITGB1
16	**ETARACIZUMAB**	ITGB1
17	**FIRATEGRAST**	ITGB1
18	**PF-04605412**	ITGB1
19	**GLPG-0187**	ITGB1
20	**SAN-300**	ITGB1

Drugs in bold have not been previously reported for AD patients

## Discussion

**Fig. 5:**
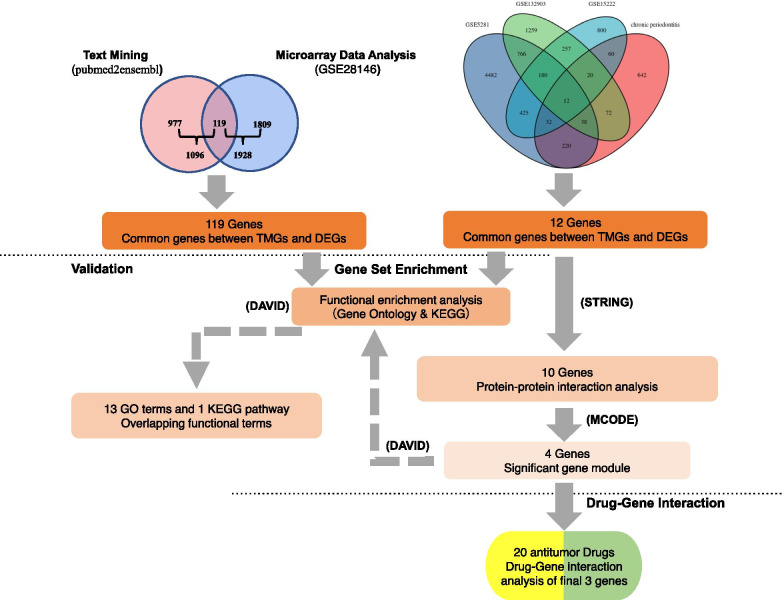
The framework of data analyses

This study explored the possible molecular biomarkers between chronic periodontitis and AD through bioinformatics analysis and data mining (Fig. 5). The results showed that through network analysis of GO, KEGG and PPI, four pivot genes (ITGB1, SPP1, CD44 and THY1) and two other genes of interest (CCL5 and BMP7) were screened out. Among them, 20 genes targeted SPP1, CD44 and ITGB1, which had therapeutic properties for AD. Moreover, after verification via the GSE28146 cohort, the only overlapping KEGG term "ECM-receptor interaction" was obtained.

In many epidemiological studies, in addition to the reported involvement of inflammatory mediators in chronic periodontitis and CHD/type-II DM, our study also found that chronic periodontitis may be the result of the gradual deterioration of neuronal function during aging. Therefore, a new potential treatment method for preventing the progression of AD has emerged: delaying or preventing chronic inflammatory diseases. However, at present, the pathogenesis and effective treatment of chronic periodontitis for cognitive decline remain unclear. Hence, it is imperative to explore the molecular mechanism of cognitive decline after chronic periodontitis to determine efficient biomarkers and effective approaches for the diagnosis, monitoring, and treatment of patients.

To obtain more reliable experimental results, our validation with a separate GSE28146 cohort revealed the only overlapping term in KEGG: "ECM receptor interaction". ECM receptors are composed of many structural and functional macromolecules, including collagen, laminin, and fibronectin (FN), especially FN [23]. At the same time, ECM receptor interactions play an important role in the microenvironmental pathways that balance the structure and function of cells and tissues. Previous reports have confirmed the role of the ECM receptor interaction pathway(s) in many cancers such as breast cancer [24], glioblastoma [25], prostate cancer [26], and colorectal cancer [27]. Unfortunately, there is no clear report about whether ECM receptor interaction is involved in the occurrence and development of chronic periodontitis and AD.

It is critical to point out that Integrin β1 (ITGB1) constitutes a prevalent gene in most of the rich KEGG pathways in AD. Additionally, the ITGB1 gene comprised one of the hub genes uncovered by the PPI network. ITGB1 is one of the most common integrin heterodimer subchains. The bi-directional signaling of ITGB1, as well as cross-talking with other cellular receptors, has been shown to play an important role in survival, cell adhesion, differentiation and proliferation [28]. Previous research has illustrated that ITGB1 plays an indispensable role in the survival and metastatic potential of lung, breast, and colon tumors [29–34]. At the same time, ITGB1 has been found to promote tumor resistance to anti-cancer drugs such as bevacizumab, erlotinib and gefitinib [35–38].

Secreted phosphoprotein 1 (SPP1) is a secreted glycophosphate protein with a wide range of functions and is also known as osteopontin, which plays an indispensable role in B cell-triggered cellular immunity [39, 40]. At the same time, it plays a significant role in numerous autoimmune diseases, e.g., rheumatoid arthritis, systemic lupus erythematosus, and multiple sclerosis [41]. Studies have shown that SPP1 levels in pyramidal neurons in the hippocampus of AD patients are significantly elevated [42].

Thymus cell antigen 1 (Thy1), alias cluster differentiation (CD) 90, which is expressed in the cell membranes of all types of cells, is a glycoprotein anchored to glycophosphatidylinositol [43]. It plays an indispensable role in cell–cell and cell–matrix interactions [44]. THY has been proven to be a cancer marker [45], and it has been found that high expression of THY1 is linked to poor prognosis in individuals with extrahepatic cholangiocarcinoma [46] and lung cancer patients [47].

CD44 is a member of the glycoprotein family. It is an inflammation-related gene that encodes widely distributed alternatively spliced cells. The glycoprotein is related to inflammation-related neuronal damage. Previous studies have shown that CD44's involvement in the pathological process of AD [48–50] may be related to its adhesion and migration in immune cells [51] and microglia [52]. Interestingly, in the study by Velez et al., it was found that the CD44 gene is specifically associated with AD, and it has been confirmed that CD44 is closely related to the age at onset of AD [53].

In addition to the above four target genes, we also found two more interesting genes, CCL5 and BMP7.

Chemokine (C–C motif) ligand 5 (CCL5), is a chemokine that can be produced by a variety of cells. CCL5 can help white blood cells enter the inflammatory area through endothelial cells [54], thereby indirectly participating in the inflammatory response. Therefore, studies have shown that after periodontitis and periodontitis treatment, the concentration of CCL5 in the blood of patients remains at a high level [55, 56]. Compared with cognitively healthy subjects, AD patients have lower CCL5 expression [57, 58]. However, in the study of Marksteiner et al. [59], CCL5 levels are higher in AD patients. In addition, the results of Soares et al. [60] found that there was no difference in the protein level of CCL5 between AD and the control group.

Recent studies have found that Bone morphogenetic protein 7 (BMP7) can be produced in the salivary glands of mice [61]. Although there is no clear report on whether BMP7 is related to periodontitis and AD in the current study, related studies have proved that BMP7 be related to a variety of tumors, such as colorectal cancer [62], breast cancer [63], and prostate cancer [64].

Among the 20 drugs discovered, 7 drugs (Calcitonin, Wortmannin, Gentamicin, Tacrolimus, Progesterone, Gentamicin, and Hyaluronan) have been reported to have certain experimental and clinical benefit for the treatment of AD. This shows that our GEO cohort based on big data the analysis has certain value for the potential treatment of AD. The remaining 13 drugs have not been found to be related to the treatment of AD and can be used as potential target drugs for AD. These include ASK-8007, Alteplase, Bivatuzumab, Hyaluronate sodium, Abituzumab, Volociximab, Natalizumab, Intetumumab, Etaracizumab, Firategrast, PF-04605412, GLPG-0187, and SAN-300.

## Conclusions

By employing a sequence of bioinformatics tools for gene expression profiling, we established the core function of key candidate genes, including ITGB1, SPP1, CD44, THY1, CCL5, and BMP7, and the enriched signaling cascades constituting the ECM-receptor interaction pathways in the molecular modulation network of cognitive decline via integrated bioinformatic analysis. Through the above results, we found that there may be a significant correlation between chronic periodontitis and AD. This provides a prospective target for the diagnosis and clinical treatment of AD in patients with chronic periodontitis in the future. However, in vitro and in vivo studies should be conducted to verify our findings.

## Data Availability

Publicly available datasets were analyzed in this study. This data can be found at GEO data repository (https://www.ncbi.nlm.nih.gov/geo/) and include the accession numbers: GSE5281, GSE15222, GSE132903 and GSE28146.

## Abbreviations

AD: Alzheimer's disease
DEGs: Differentially expressed genes
GEO: Gene Expression Database
NCBI: National Center for Biotechnology Information
STRING: Search Tool for the Retrieval of Interacting Genes
DGIDB: Drug gene interaction database
FN: Fibronectin
Itgb1: Integrin β1
SPP1: Secreted phosphoprotein 1
Thy1: Thymus cell antigen 1
